# Tree seed rain and seed removal, but not the seed bank, impede forest recovery in bracken (*Pteridium aquilinum* (L.) Kuhn)‐dominated clearings in the African highlands

**DOI:** 10.1002/ece3.3944

**Published:** 2018-04-02

**Authors:** Fredrick Ssali, Stein R. Moe, Douglas Sheil

**Affiliations:** ^1^ Faculty of Environmental Sciences and Natural Resource Management Norwegian University of Life Sciences Ås Norway

**Keywords:** Afro‐tropical forest, Bwindi, forest‐bracken edge, invasive species, *Pteridium aquilinum*, recruitment limitation, seed rain, seed removal, soil seed bank

## Abstract

Considerable areas dominated by bracken *Pteridium aquilinum* (L.) Kuhn occur worldwide and are associated with arrested forest recovery. How forest recovery is impeded in these areas remains poorly understood, especially in the African highlands. The component processes that can lead to recruitment limitation—including low seed arrival, availability and persistence—are important determinants of plant communities and offer a potential explanation for bracken persistence. We investigated key processes that can contribute to recruitment limitation in bracken‐dominated clearings in the Bwindi Impenetrable National Park, Uganda. We examined if differences in seed rain (dispersal limitation), soil seed bank, or seed removal (seed viability and persistence) can, individually or in combination, explain the differences in tree regeneration found between bracken‐dominated areas and the neighboring forest. These processes were assessed along ten 50‐m transects crossing the forest–bracken boundary. When compared to the neighboring forest, bracken clearings had fewer seedlings (bracken 11,557 ± 5482 vs. forest 34,515 ± 6066 seedlings/ha), lower seed rain (949 ± 582 vs. 1605 ± 335 tree seeds m^−2^ year^−1^), comparable but sparse soil seed bank (304 ± 236 vs. 264 ± 99 viable tree seeds/m^2^), higher seed removal (70.1% ± 2.4% vs. 40.6% ± 2.4% over a 3‐day interval), and markedly higher rodent densities (25.7 ± 5.4 vs. 5.0 ± 1.6 rodents per 100 trapping sessions). Camera traps revealed that rodents were the dominant animals visiting the seeds in our seed removal study. *Synthesis*: Recruitment limitation contributes to both the slow recovery of forest in bracken‐dominated areas, and to the composition of the tree species that occur. Low seed arrival and low persistence of unburied seeds can both explain the reduced density of seedlings found in bracken versus neighboring forest. Seed removal, likely due to rodents, in particular appears sufficient to constrain forest recovery and impacts some species more severely than others.

## 1 INTRODUCTION

The role of clearings dominated by bracken fern *Pteridium aquilinum* (L.) Kuhn in retarding forest recovery is a worldwide concern. Areas dominated by such fern thickets occur on every continent except Antarctica and cover thousands of square kilometers of previously forested habitat (Holm, Doll, Holm, Pancho, & Herberger, 1997; Marrs & Watt, 2006; Verdcourt, 1999). Temperate studies have suggested that bracken, *Pteridium aquilinum*, interferes with forest regrowth due to the effects of shade, pathogens, allelopathy, deep litter or perhaps seed removal (den Ouden, 2000; Dolling, 1996; Facelli & Pickett, 1991; Gaudio et al., 2011; Ghorbani, Duc, Mcallister, Pakeman, & Marrs, 2007; Priewasser, 2013). However, how these factors and mechanisms might operate singly or in combination has seldom been examined especially in equatorial Africa (but see Ssali, Moe, & Sheil, 2017).

Recruitment limitation—failure of a species to arrive and establish—is an important determinant of plant communities. This limitation can arise from multiple processes including low seed production, seed dispersal, or persistence (Marques & Burslem, 2015). Crucially, little is known about the role of recruitment limitation in explaining bracken persistence although previous temperate studies have shown that seed addition can sometimes increase tree regeneration in bracken‐dominated areas (Ghorbani et al., 2007; Pakeman, Le Duc, & Marrs, 2002). Studies elsewhere have shown that recruitment failure in deforested areas can be the result of seed limitation and/or establishment limitation (see, e.g., Muscarella et al., 2013; van Eck, de Steeg, Blom, & de Kroon, 2005). Here we focus on the early life‐history stages where recruitment limitation can be manifested, that is, seed arrival, seed persistence (and viability), and seed removal.

Successful establishment requires the arrival and persistence of viable seeds, germination, and seedling survival. Our initial exploration showed that bracken‐dominated areas are persistent and indicated that species with longer seed dormancy and greater growth potential are better suited to establish in bracken than are species lacking these characteristics (Ssali et al., 2017). However, how seed limitation and establishment limitation, that is, seed rain, soil seed bank, and seed removal, influence tree regrowth in bracken clearings remains poorly understood despite the existing literature (from temperate and neotropical regions, see Marrs & Watt, 2006; Priewasser, 2013; Royo & Carson, 2006).

Dispersal limitation (i.e., low seed arrival) in open areas adjacent to tropical forest has attracted much attention (e.g., Barnes & Chapman, 2014; Duncan & Chapman, 1999; Wijdeven & Kuzee, 2000). Notably, Duncan and Chapman (1999) found that seed rain in deforested habitats decreases rapidly with distance from the forest. The nonzoochorous tree species, especially early successional species, tend to produce numerous small seeds that can be transported by wind, but most tropical forest tree species depend on frugivorous animals for dispersal (Ghazoul & Sheil, 2010). Observations in the neotropics have indicated that the seed rain of animal‐dispersed species is influenced by the availability of food and of perching sites (e.g., Saavedra, Hensen, & Schleuning, 2015). Studies in African forests have also found that the arrival of large‐seeded species depends on large mammals including elephants and primates (Babweteera, Savill, & Brown, 2007; Beaune, 2015; Gross‐Camp, Masozera, & Kaplin, 2009). Indeed, in the forest adjacent to bracken clearings in the Bwindi Impenetrable National Park in Uganda, animal‐dispersed species comprise approximately 80% of the common tree species (Ssali et al., 2017).

Following dispersal, the seeds may be incorporated into the soil where they can persist for months before germinating (Ghazoul & Sheil, 2010). However, temperate studies have found that bracken fronds and litter impede the incorporation of seeds into the soil (Ghorbani, Le Duc, McAllister, Pakeman, & Marrs, 2006; Pakeman & Hay, 1996). Further, temperate and neotropical studies have found that the soil seed bank in bracken may be negatively affected by predation, allelopathy, or trampling by large mammals (De Jesus Jatoba et al., 2016; Ghorbani et al., 2007). Furthermore, species with longer seed dormancy may possess greater potential to survive bracken interference and can thus dominate the seed bank in bracken (Ssali et al., 2017).

Seed removal studies in the tropics have shown that few seeds persist if they arrive in thick undergrowth (Blackham & Corlett, 2015; Razafindratsima, 2017). Typically, the main agents of seed removal are rodents (Hulme, 1998), although other animals can be involved, for example, insects (Gallegos, Hensen, & Schleuning, 2014), birds (Cordeiro, Ndangalasi, McEntee, & Howe, 2009), and large mammals (Beaune, Bollache, Fruth, & Bretagnolle, 2012; Feer, 1995). Once removed, seeds may experience different fates including predation or being scatter‐hoarded by rodents which may then fail to retrieve them (Aliyu, Adamu, Moltchanova, Forget, & Chapman, 2014; Forget, 1991; Janzen, 1970; Traveset, Heleno, & Nogales, 2014).

In the Bwindi Impenetrable National Park (“Bwindi”) in Uganda, forest recovery in bracken‐dominated clearings is slow (Ssali et al., 2017). Our hypothesis here is that the limited forest recovery in bracken compared to forest is related to differences in recruitment limitation (namely seed arrival, persistence, and predation). Thus, in this study, using a series of integrated field studies, we explored the role of recruitment limitation in explaining the difference in regeneration between bracken and neighboring forest environments by examining seedling densities, seed rain, soil seed bank, and seed removal rates (and rodent densities). Given that these limitations will favor or filter species in a manner that may shed light on the underlying processes, we are interested in both the implications for overall woody regeneration and the differences that arise among individual species.

## 2 MATERIALS AND METHODS

The study was conducted in the Bwindi Impenetrable National Park (henceforth “Bwindi”) a UNESCO World Heritage site in South‐West Uganda. Bwindi is located at 0°53′–1°08′ S, 29°35′–29°50′ E near the equator and spans a wide range of elevations (1,160–2,607 m asl). The park's main vegetation is classified as moist lower montane forest (Hamilton, 1982; Howard, 1991). The climate is tropical with two rainfall peaks from March to May and September to November. Annual rainfall ranges from 1,130 to 2,390 mm and the mean temperature ranges between 7 and 29°C. The driest months are December‐January and July‐August. Bwindi is relatively rich in species owing to high rainfall, range of elevations, and likely proximity to Pleistocene refugia (Hamilton, 1982).

Bwindi is rugged, steep, and divided by cliffs, with a remarkably dense understorey. The forest canopy is open in many areas due in part to extensive clearings dominated by bracken fern *Pteridium aquilinum* (Ssali et al., 2017). The clearings have been created by past fires, landslides, timber cutting, and cultivation (Babaasa, Eilu, Kasangaki, Bitariho, & McNeilage, 2004; Olupot, Barigyira, & Chapman, 2009; Ssali et al., 2017). Other understorey species dominate other areas both in the continuous forest and in the open clearings. Many of the common understorey species form extensive stands and flower only every few decades before seeding and dying en masse. Examples include the African mountain bamboo *Yushania alpina* (K. Schum.) Lin. and various Acanthaceae including the thicket‐forming *Mimulopsis solmsii* Schweinf. and *Mimulopsis arborescens* C.B. Clarke. Along with other understorey species, these monocarpic species are key food plants for mountain gorillas *Gorilla beringei beringei* Matschie (Sheil, 2012).

Our field observations were conducted between June 2015 and December 2016. We selected 10 bracken‐dominated clearings accessible from the Institute of Tropical Forest Conservation (ITFC)—a research station at Ruhija (2,355 m asl). Within each clearing, we established one 50 × 5‐m transect across the forest‐bracken boundary with the 25‐m mid‐point at the forest edge and 25 m extending one way into the bracken and the other into the forest. We recorded all woody stems and the number of bracken fronds in eleven 1‐m^2^ quadrats established 5‐m apart along each of the 10 transects. We also measured slope (° using a clinometer) in the middle and at each end of the transect and interviewed local informants who had worked in these forests for many years about the history of each site.

We assessed the seed rain by placing 11 seed traps at 5‐m intervals along each transect. Each trap consisted of a high‐density polyethylene funnel (diameter = 27 cm) mounted on wooden poles 40 cm above the ground with the spout resting on a small basket which contained a collection bag. The traps were visited every 2 weeks and the trapped material collected and then processed at the research station. Seeds were lost occasionally (eight of 4,422 trap visits) when the traps were knocked down by African bush elephant *Loxodonta africana* Blumenbach and when the collection bags were removed (possibly by L'Hoest's monkey *Cercopithecus l'hoesti* P. Sclater or Olive baboon *Papio anubis* Lesson). Seeds were identified at ITFC with references (Hamilton, 1991; Katende, Birnie, & Tengnäs, 1995), voucher specimens in ITFC's herbarium or by identifying parent trees where seeds were still attached. Nomenclature follows Global Plants (http://plants.jstor.org/).

To assess the soil seed bank, along each transect we collected and combined five 10.2‐cm‐wide and 10‐cm‐deep soil cores 1‐m apart in the forest (−25 m), at the edge (0 m) and in bracken (25 m). The soil samples were kept in paper bags and processed at the ITFC research station. Each soil sample was spread on a fine mesh (2 mm) and washed to separate soil from seeds. The washed samples, which mainly comprised fibrous matter and seeds, were spread in trays and placed in a transparent polythene shelter—a well‐illuminated environment where viable seeds were germinated. In addition, trays containing seed‐free sand were placed among the sample trays to control for any dispersal of seeds into the germination shelter. All the trays were kept moist and were watered and checked for emerging seedlings every 2 days. We also mixed the remaining material every month to ensure the remaining seeds were exposed to light. No seedlings emerged from the trays containing sand in the 6 months of the study. Seedlings were identified by ITFC's botanical staff and removed as they emerged.

We assessed seed removal by placing wooden trays, each with 13 different seed species, in eleven above‐ground locations 5‐m apart along each of the 10 transects. Litter was removed to make space for the seed stations. The 13 species were the trees *Strombosia scheffleri*,* Chrysophyllum pruniforme*,* Allophylus abyssinicus*,* Macaranga capensis*,* Neoboutonia macrocalyx*,* Polyscias fulva*,* Clutia abyssinica*,* Faurea saligna*,* Psychotria mahonii,* and *Olinia rochetiana,* and also the crops *Oryza glaberrima*,* Arachis hypogaea,* and *Zea mays*. The first 10 are common near the research station and regularly produce seeds between June and August (ITFC unpublished data), while the last three are common crops in the villages neighboring the park. All tree seeds were stored in paper bags and placed out in their fruits except for seeds of *Chrysophyllum pruniforme* which were manually separated from their fleshy arils. We recorded the number of seeds removed after 3 days over 10‐census periods carried out in June‐August 2016 (*n* = 1,430 seeds). During each census, we replaced any removed or damaged seeds with fresh ones.

To assess rodents, we placed Sherman live traps (H. B. Sherman Inc., Tallahassee, Flor. 8 × 8 × 23 cm) at 5‐m positions along the 10 transects. Traps were set on the ground. For bait, we combined sweet bananas, maize flour, and roasted powdered groundnuts and left them to ferment overnight following Isabirye‐Basuta and Kasenene (1987) and Kasangaki, Kityo, and Kerbis (2003). The traps were inspected twice daily for five consecutive days (late July and early August 2016): early in the morning for nocturnal species and late in the evening for diurnal species. Species and weight were recorded before release. We also attempted to identify the animals implicated in seed removal by deploying camera traps (Reconyx model RM45; http://www.reconyx.com) preset with a 1‐s delay between subsequent images in two locations along seven transects during the last 3 days of the seed removal study. We count and analyse “events” as one or more sequences of images separated by no more than one minute. Nomenclature follows Wilson and Reeder (2005).

### 2.1 Analyses

Woody plants were categorized as large trees (dbh ≥ 10 cm), saplings (dbh 2–9.9 cm), large seedlings (basal diameter <2 cm and >30 cm tall), and small seedlings (<30 cm tall). We examined stem abundance, seed rain, soil seed bank, and seed removal in three segments of the 50 × 5‐m transects, that is, “forest” (ten 83.5‐m^2^ plots), “edge” (ten 83.5‐m^2^ plots), and “bracken” (ten 83.5‐m^2^ plots).

To examine whether seedling densities, seed rain, and soil seed bank differed between forest and bracken, we calculated the degree of dissimilarity between the abundance of each common woody species in bracken versus that in the forest using a modified Bray‐Curtis dissimilarity index (Bray & Curtis, 1957):

$$y_{B,F}=\left[\frac{\left(m_{\mathrm{iB}}+0.5\right)-\left(m_{\mathrm{iF}}+0.5\right)}{\left(m_{\mathrm{iB}}+0.5\right)+\left(m_{\mathrm{iF}}+0.5\right)}\right]\times \;100$$

where *y*_*B,F*_ denotes the dissimilarity index, *m*_*iB*_ the abundance of the *i*th common woody species in bracken, and *m*_*iF*_ the abundance of the *i*th common woody species in the forest. We obtained the dissimilarity index by subtracting the number of seeds per‐species in each transect segment of forest from the corresponding number of seeds in bracken and divided the outcome by the sum of seeds for the forest and bracken transect segments combined. We added a half to the number of seeds in both forest and bracken transect segments to avoid the occurrence of zero values in the denominator and multiplied the result by 100. All analyses used R (R Core Team, 2016).

## 3 RESULTS

### 3.1 Clearings, bracken, and seedlings

**Figure 1:**
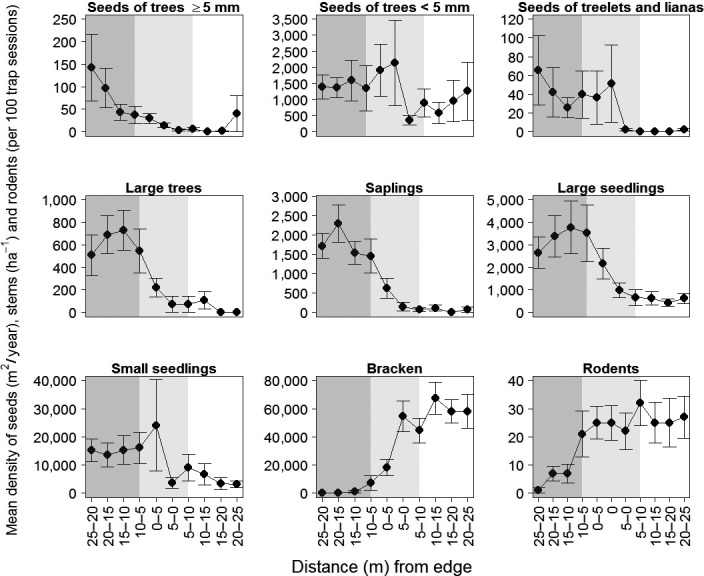
Seed rain density (seeds m^−2^ year^−1^ ± 1 *SE*), density of trees (stems/ha ±1 *SE*) and density of rodents (per 100 trap sessions ±1 *SE*) plotted relative to the edge between the forest and bracken. Distance classes are successive 25 m^2^ 5 m‐transect segment with the considered “forest” marked dark gray, the “edge” in lighter gray, and the bracken‐dominated area unshaded. Treelets are here defined as free standing woody plants with one or more stems that seldom have stem diameters or overall height exceeding 5 cm or 5 m respectively (see Appendix S1 for a full species list)

Our 10 transects intersected bracken‐dominated clearings ranging in area from 0.1 ha to 2.5 ha and with slopes from 12 to 35°. According to local informants, the 10 clearings had been affected by fire (one had last burned in 1998 and the other nine in the 1980s). Of the 10 clearings, eight had been impacted by past timber cutting and extraction, two by landslides and two by other human activity (settlements and related). On average (±1 *SE*), bracken density in the clearings was 3.0 ± 0.3 fronds/m^2^. The bracken‐dominated area typically had fewer seedlings, saplings, and large trees as well as markedly higher rodent density than the nearby forest (Figure 1). When comparing trees at different life stages, the ratio of small to large seedlings in forest versus bracken was similar (1.187 vs. 1.089, i.e., 0.99 times), that from large seedlings to saplings was about nine times (0.093 vs. 0.01, i.e., 9.37 times) and from saplings to large trees was again similar (0.397 vs. 0.4, i.e., 1.09 times).

### 3.2 Seed rain

We collected 12,997 seeds of 39 woody species (trees, treelets, and woody climbers) over 18 months from the seed traps. The seeds comprised 34 woody species (*n* = 5,536 seeds) in the forest, 29 woody species (*n* = 4,251 seeds) at the edge, and 20 woody species (*n* = 3,210 seeds) in bracken (Appendix S1). Trees comprised 97.3% of these seeds in the forest, 97.7% at the edge, and 99.9% in bracken. The remainder of the seeds belonged to woody climbers and treelets (i.e., free standing woody plants with one or more stems that seldom have stem diameters or overall height exceeding 5 cm or 5 m, respectively). On average (±1 *SE*) seed rain density (tree seeds m^−2^ year^−1^) was 1604.7 ± 334.6 in the forest, 1625.3 ± 708.1 at the edge, and 948.5 ± 581.8 in bracken. The average abundance of seeds declined significantly with distance from the forest into bracken‐dominated clearings, except for small‐seeded species (i.e., for larger (≥5 mm) seeds of trees: Kendall's correlation, τ = −0.67, *p* = .003; for smaller (<5 mm) seeds of trees: τ = −0.27, *p* = .283; seeds of treelets and lianas: τ = −0.62, *p* = .009; *n* = 11 in all cases; see Figure 1).

**Figure 2:**
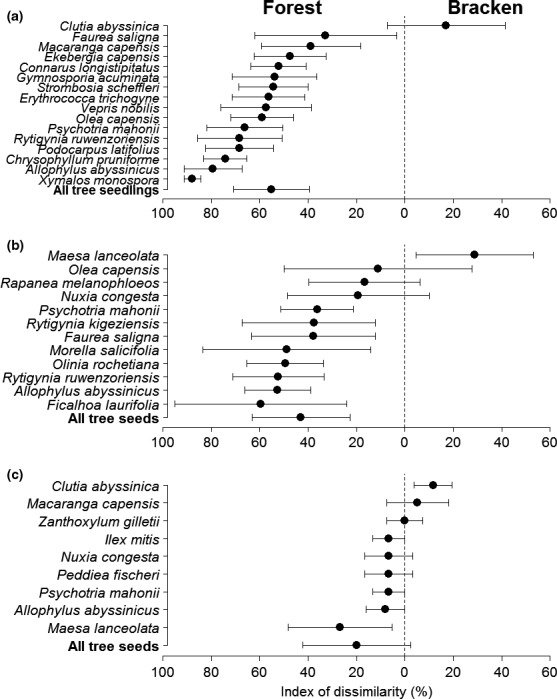
Forest versus bracken dissimilarity index (absolute values ±1 *SE*) of common tree species in the (a) seedlings, (b) seed rain, and (c) soil seed bank. The selected common tree species had (a) five or more occurrences in the seedling population, (b) four or more occurrences in seed rain, and (c) two or more occurrences in soil seed bank in the forest and/or bracken

At species level, seed density declined significantly with distance from the forest interior into bracken clearings for nine of the 16 most common woody species (see Appendices S2 and S3). In addition, of the 12 tree species with four or more occurrences in the forest and bracken combined (edge excluded) seven species had significantly greater relative abundance in the forest while only one species, *Maesa lanceolata*, had significantly greater relative abundance in bracken (Figure 2a). We found a significant positive relationship between seed rain dissimilarity index and the dissimilarity index of seedlings (Kendall's rank correlation, τ = 0.3, *n* = 12, *p* = .025) and no significant correlation between seed rain dissimilarity index and mean seed size of common tree species (τ = 0.12, *n* = 12, *p* = .582). In bracken, seed rain density of four of seven common tree species was positively correlated with seedling density but only *Nuxia congesta* had a significant correlation (τ = 0.75, *n* = 10, *p* = .013).

### 3.3 Soil seed bank

A total of 6,953 seeds germinated and emerged from the soil samples (and none from the controls). Five hundred and fifty were seedlings belonging to a total of 15 woody species, 5,905 plants belonged to nonwoody species while 498 could not be identified (Appendix S1). Of the 15 woody species, 12 emerged from soil collected in the forest, nine species from the edge, and seven from bracken. On average, the density of viable seeds in the soil (tree seeds/m^2^) was 264.4 ± 99.3 in the forest, 181.2 ± 65.9 at the edge and 303.5 ± 236.1 in bracken. Seeds of the treelet *Clutia abyssinica* were significantly more abundant in bracken (dissimilarity index = 11.7 ± 7.8) while *Maesa laceolata* was significantly more abundant in the forest (dissimilarity index = 26.7 ± 21.3; Figure 2b, despite a considerably greater abundance of *Maesa laceolata* in bracken than in the forest at one transect; see Appendix S1). We found no significant correlation between seed bank dissimilarity index and the dissimilarity index of seedlings of common tree species (τ = 0.08, *n* = 9, *p* = .798). In bracken, seed bank density of three of four common species was positively correlated with seedling density but none reached significance (all *p* values >.1).

### 3.4 Seed removal

**Figure 3:**
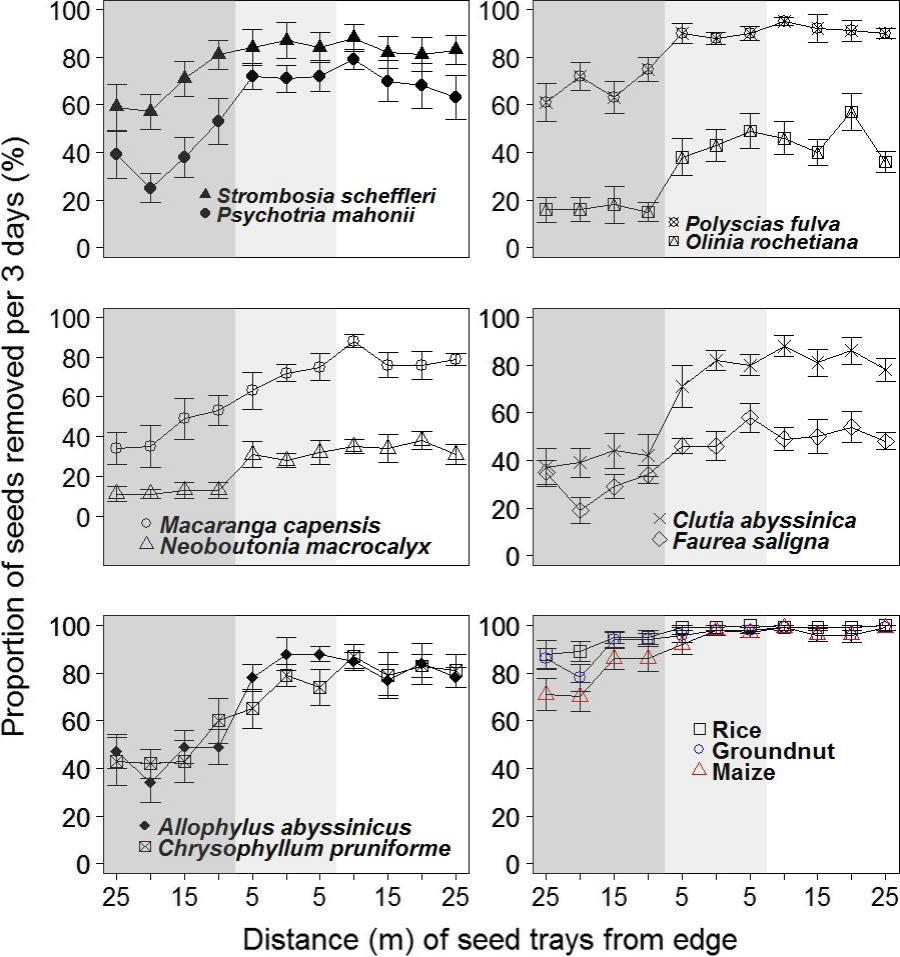
Proportion of seeds removed by location. Trays are placed every 5‐m starting in the forest and ending in bracken. The area termed “forest” is dark gray, “edge” is gray, and the bracken‐dominated area is unshaded. The forest species (in bold italic) are common tree species within the park that had a substantial number of seeds during the census while the grain crops (in bold) were used as a way to assess removal for attractive seeds

The mean proportion of seeds of woody species removed over 3 days was 40.6% ± 2.4% in the forest, 67.5% ± 2.4% in the edge and 70.1% ± 2.4% in bracken. *Polyscias fulva* had the highest proportion of seeds removed in both the bracken area (92.0% ± 2.4%) and forest (67.7% ± 2.4%) per 3 days followed by *Strombosia scheffleri* with removal rates of 83.5 ± 2.4% and 67.0% ± 2.4% in bracken and the forest, respectively (Figure 3). *Neoboutonia macrocalyx* was removed the least with removal rates of 34.5% ± 2.4% and 12.0% ± 2.4% per 3 days followed by *Olinia rochetiana* with removal rates of 44.7% ± 2.4% and 16.2% ± 2.4% in bracken and the forest, respectively. All seeds of woody species included in the study had lower rates of removal than all three grain crops, which also showed high rates of removal in the forest (see Figure 3). We found a significant positive correlation between removal rates of woody species in forest and the removal rates of woody species in bracken (τ = 0.87, *n* = 10, *p* = .0001), between the removal rates in forest and at the edge (τ = 0.82, *n* = 10, *p* = .0004), and between the removal rates at the edge and in bracken (τ = 0.82, *n* = 10, *p* = .0004). We detected no significant correlation between mean seed size and seed removal per 3 days of woody species in forest (τ = 0.1, *n* = 10, *p* = .728), at the edge (τ = −0.02, *n* = 10, *p* = 1), in bracken (τ = 0.16, *n* = 10, *p* = .601), and in all segments combined (τ = 0.06, *n* = 10, *p* = .862).

### 3.5 Rodent captures

A total of 217 rodents were captured on the 10 transects in 1,100 trapping sessions (550 days, morning and evening). Captures occurred approximately five times more frequently in the bracken than in the forest (Table 1). We found a significant positive relationship between rodent captures and the density of bracken fronds (Kendall's rank correlation, τ = 0.89, *n* = 10, *p* = .001). Assuming that captures are proportional to densities we estimated rodent biomass to be around six times higher in bracken than in the forest due to the large species *Lophuromys flavopunctatus* and *Lophuromys woosnami* representing the majority of the rodent biomass in bracken and the smaller *Praomys* spp representing about half of the rodent biomass in the forest (Table 1). The images from the camera traps selectively employed in seven transects (*n* = 544 images) were not always readily identified (either the animal or its activity) due to problems of visibility (and activation of the motion detector particularly in the thicker vegetation outside the forest: 62 of 211 images in the forest were clearly discernible compared to only 40 of just 202 images in bracken). While bracken‐dominated sites averaged 65% fewer images of rodents removing seeds than the forest sites, these same bracken sites had a 78% higher rate of seed removal (Appendix S4). The number of clear rodent “events” (sequences of observations of specific species separated by less than one minute) and proportion of seeds removed were not significantly correlated (τ = 0.1, *n* = 10, *p* = .717). Nonetheless, these “events” indicated that most of the species captured in the live traps along with other rodents including large ones like *Cricetomys emini* and *Funisciurus carruthersi* that were too large to trap, visited locations where they could remove seeds (Table 2).

**Table 1:** Abundance and biomass of rodents (mean ± 1 *SE*) per 100 trapping sessions in the forest, edge, and bracken and their feeding classification based on Happold (1996)

Species	Rodent abundance			Rodent biomass (g)			Feeding classification
	Forest	Edge	Bracken	Forest	Edge	Bracken	
*Lophuromys flavopunctatus* Thomas, 1888	0	7.0 ± 2.7	12.3 ± 4.9	0	290.0 ± 99.3	479.0 ± 186.5	Omnivore
*Lophuromys woosnami* Thomas, 1906	0.3 ± 0.3	3.7 ± 1.9	6.0 ± 1.7	12.0 ± 12.0	140.1 ± 70.3	222.3 ± 63.0	Omnivore
*Praomys* spp.	2.3 ± 1.1	2.3 ± 0.9	4.7 ± 1.9	77.0 ± 42.7	84.0 ± 31.1	159.7 ± 63.0	Granivore
*Hybomys univittatus* (Peters, 1876)	1.3 ± 0.7	7.3 ± 2.7	1.3 ± 0.7	51.0 ± 27.6	295.7 ± 108.6	65.7 ± 35.8	Omnivore
*Hylomyscus vulcanorum* Lönnberg & Gyldenstolpe, 1925	1.0 ± 0.7	2.0 ± 0.7	0.7 ± 0.4	15.7 ± 11.3	45.7 ± 17.7	25.7 ± 18.2	Granivore
*Praomys jacksoni* (de Winton, 1897)	0	1.3 ± 0.7	0	0	59.0 ± 32.3	0	Granivore
*Mus bufo* (Thomas, 1906)	0	0.3 ± 0.3	0.3 ± 0.3	0	4.0 ± 4.0	1.7 ± 1.7	Granivore
Unknown species (escaped)	0	0	0.3 ± 0.3	–	–	–	
All rodent species	5.0 ± 1.6	24.0 ± 4.2	25.7 ± 5.4	155.7 ± 62.4	918.4 ± 158.4	979.4 ± 195.4	

**Table 2:** Number of identified rodent “events” and estimated rodent biomass (mean ± 1 SE) in the forest, edge and bracken

	Events over 3 days			Estimated rodent biomass (g) over 3 days		
	Forest	Edge	Bracken	Forest	Edge	Bracken
N cameras over 3 days	5	4	5			
Species						
* Lophuromys* spp.	1	6	7	7.8 ± 7.8	58.4 ± 46.4	54.5 ± 45.4
* Hybomys univittatus* (Peters, 1876)	0	0	3	0	0	24.5 ± 24.5
* Praomys* spp.	23	8	32	186.1 ± 105.2	80.9 ± 49.5	258.9 ± 182.1
* Hylomyscus vulcanorum* Lönnberg & Gyldenstolpe, 1925	11	5	0	52.1 ± 52.1	29.6 ± 29.6	0
* Cricetomys emini* Wroughton, 1910	0	0	1	0	0	247.0 ± 247.0
* Funisciurus carruthersi* Thomas, 1906	13	0	0	1662.7 ± 1023.2	0	0
Unidentified rodents	2	15	4	–	–	–
All rodent species	50	34	47	318.1 ± 167.3	28.2 ± 3.2	97.5 ± 38.8

### 3.6 Role of woody cover and perches

We examined if the local abundance of trees influenced key variables (potential sources of seeds and perches for birds and bats). We compared the local abundance of large trees and basal area with seedlings, seed rain and soil seed bank in the forest, edge, and bracken (Appendix S1). None of these relationships achieved significance although the correlation between tree basal area in all segments and seed rain in bracken and that between basal area in bracken and seed rain in bracken were borderline (*p* < .1).

## 4 DISCUSSION

### 4.1 Synthesis and overview

Our data show that when compared to the neighboring forest, bracken thickets not only had fewer seedlings, saplings, and large trees but also received fewer seeds in the seed rain and any unburied seeds were more likely to be removed (reduced persistence). Both the limited seed arrival and the low persistence of unburied seeds can limit establishment of tree seedlings in bracken‐dominated clearings. The low density of seeds in the soil seed bank, which is similar across the forest and bracken, plays no major role in generating the differences in regrowth, although it is clearly important for the local abundance of the treelet *Clutia abyssinica*. The proportion of seeds removed over 3 days was considerably higher in bracken than in the forest with rates of removal for some tree species like *Neoboutonia macrocalyx* and *Olinia rochetiana* differing by a factor of two or three (Figure 3). This marked difference in seed removal will be higher when compounded over longer periods. The per‐species relative abundance of seedlings in the forest versus bracken had a significant positive relationship with the per‐species relative abundance of the seed rain but had no relationship with the per‐species relative abundance of viable seeds in the soil. The low persistence of seeds in bracken is likely related to the high densities of rodents. Taken together, these findings indicate that recruitment limitation contributes to the limited establishment of trees in bracken, and that these factors differ among species and thus influence species composition.

When comparing forest and bracken, we found that tree seedling densities were ~ 3.0 times higher in the forest than in bracken (34,515 ± 6066 vs. 11,557 ± 5482 seedlings/ha). We also found roughly similar stem‐size density ratios in both forest and bracken in our 10 transects and a markedly lower ratio of saplings to large seedlings in bracken than in forest. The low abundance of seedlings appears to be largely a result of limited recruitment or poor survival rather than rapid development as the abundance of more mature life stages is lower or similar to those seen in forest (see also Ssali et al., 2017). The difference for tree seed rain was 1.7 times (forest 1605 ± 335 vs. bracken 949 ± 582 vs. tree seeds m^−2^ year^−1^), and for the seed bank was 0.9 times (264 ± 99 vs. 304 ± 236 viable tree seeds/m^2^). The difference for seed persistence rates was 2.0 times (59.4% ± 2.4% vs. 29.9% ± 2.4% per 3‐day period). These numbers indicate that tree seed arrival and low tree seed persistence can explain the limited recovery of forest vegetation in bracken.

When we examined tree species (the most common plus those used in the seed removal study, see Table 3), we found similar results in most cases although there were some exceptions. Three species *Nuxia congesta*,* Psychotria mahonii,* and *Maesa lanceolata* had higher seed rain in bracken than in the forest, for example. *Nuxia congesta* produces small hairy capsules containing many seeds that are easily transported by wind (Katende et al., 1995) while *Psychotria mahonii* and *Maesa lanceolata* produce numerous small fleshy fruits which are eaten and dispersed by primates or birds (Graham, Moermond, Kristensen, & Mvukiyumwami, 1995; Gross‐Camp et al., 2009; Sun, Ives, Kraeuter, & Moermond, 1997). The three most abundant tree seedlings that occurred in bracken were *Faurea saligna*,* Psychotria mahonii* and *Rytigynia ruwenzoriensis*. These species also ranked highly in the local seed rain in bracken (2nd, 3rd, and 5th, respectively) but had no seed bank. *Faurea saligna* produces small hairy nutlets that lose viability in 1 month (Katende et al., 1995) while *Rytigynia ruwenzoriensis* also produces fleshy fruits and is dispersed by animals, including primates (Rothman, Nkurunungi, Shannon, & Bryer, 2014). *Clutia abyssinica*, which has gravity dispersed seeds capable of dormancy (Katende et al., 1995; Shehaghilo, 1989), was significantly more abundant in the soil seed bank in bracken than in the forest (see Figure 2b) and was the only common woody species whose seedlings were more abundant in bracken. The link between seed rain and seedling abundance in explaining the difference between forest and bracken is further underlined by the correlation between the dissimilarity indices for the most common species.

**Table 3:** List of the most common woody species with seedlings in bracken showing their mean seedling density (stems/ha), seed rain density (seeds m^−2^ year^−1^), soil seed bank (viable seeds/m^2^) and rate of seed persistence (% per 3‐day interval)

Species	Seedlings	Seed rain	Soil seed bank	Seed persistence
(a) Forest				
*Faurea saligna* Harv.	58.4 ± 33.2	699.4 ± 271.2	0	70.8 ± 3.2
*Psychotria mahonii* C.H.Wright	68.0 ± 29.8	13.7 ± 6.1	4.9 ± 4.9	61.3 ± 6.1
*Rytigynia ruwenzoriensis* (De Wild.) Robyns	95.7 ± 31.8	15.2 ± 8.1	2.4 ± 2.4	NA
*Clutia abyssinica* Jaub. & Spach	5.7 ± 3.3	0	0	59.5 ± 6.0
*Nuxia congesta* R.Br. ex Fresen.	0	274.6 ± 137.9	14.7 ± 9.8	NA
*Croton macrostachyus* Hochst. ex Delile	0	18.1 ± 13.3	6.1 ± 6.1	NA
*Macaranga capensis* (Baill.) Sim	5.3 ± 2.0	3.2 ± 2.6	15.3 ± 12.2	57.3 ± 6.8
*Allophylus abyssinicus* (Hochst.) Radlk.	31.6 ± 9.3	88.6 ± 70.2	12.2 ± 12.2	55.3 ± 5.3
*Maesa lanceolata* Forssk.	0	0.9 ± 0.9	215.4 ± 84.6	NA
*Ekebergia capensis* Sparrm.	4.2 ± 1.7	1.8 ± 1.2	0	NA
*Strombosia scheffleri* Engl.	3.4 ± 1.6	0	0	33.0 ± 6.5
*Chrysophyllum pruniforme* Pierre ex Engl.	11.3 ± 6.7	9.4 ± 6.2	0	53.0 ± 6.4
*Neoboutonia macrocalyx* Pax	0.6 ± 0.6	21.1 ± 18.6	0	88.0 ± 1.8
*Olinia rochetiana* Juss.	0	3.8 ± 1.7	24.5 ± 24.5	83.8 ± 4.4
*Polyscias fulva* (Hiern) Harms	0	46.5 ± 45.8	0	32.3 ± 5.3
% All species in the forest	345.2 ± 60.7	1604.7 ± 334.6	264.4 ± 99.3	59.4 ± 2.4
(b) Bracken				
*Faurea saligna* Harv.	63.2 ± 52.5	130.7 ± 48.2	0	49.8 ± 3.5
*Psychotria mahonii* C.H.Wright	27.7 ± 16.5	71.6 ± 69.7	0	30.0 ± 7.0
*Rytigynia ruwenzoriensis* (De Wild.) Robyns	6.6 ± 3.8	9.6 ± 9.6	0	NA
*Clutia abyssinica* Jaub. & Spach	6.6 ± 2.6	0	3.1 ± 3.1	16.8 ± 3.9
*Nuxia congesta* R.Br. ex Fresen.	4.2 ± 2.8	686.0 ± 523.1	0	NA
*Croton macrostachyus* Hochst. ex Delile	2.4 ± 1.5	0.3 ± 0.3	0	NA
*Macaranga capensis* (Baill.) Sim	0.8 ± 0.6	1.5 ± 1.5	6.1 ± 4.0	20.3 ± 3.7
*Allophylus abyssinicus* (Hochst.) Radlk.	0.7 ± 0.5	0.6 ± 0.4	0	19.0 ± 4.9
*Maesa lanceolata* Forssk.	0.7 ± 0.7	16.7 ± 15.7	252.1 ± 233.3	NA
*Ekebergia capensis* Sparrm.	0.2 ± 0.2	0	0	NA
*Strombosia scheffleri* Engl.	0.1 ± 0.1	0.6 ± 0.6	0	16.5 ± 5.5
*Chrysophyllum pruniforme* Pierre ex Engl.	0	0.6 ± 0.6	0	17.5 ± 5.7
*Neoboutonia macrocalyx* Pax	0	0	0	65.5 ± 4.1
*Olinia rochetiana* Juss.	0	0	0	55.3 ± 4.9
*Polyscias fulva* (Hiern) Harms	0	0	0	8.0 ± 2.9
All species in bracken	115.6 ± 54.8	948.5 ± 581.8	303.5 ± 236.1	29.9 ± 2.4

Seed removal rates although generally high varied among species. For example, *Strombosia scheffleri* and *Polyscias fulva* had higher seed removal rates per 3 days both in the forest and in bracken than *Neoboutonia macrocalyx*,* Olinia rochetiana,* and *Faurea saligna*. Of these species, the only one that is found relatively frequently as a seedling in bracken is *Faurea saligna* which also appears to be a well defended and common seed in the seed rain. Overall our results suggest that species with readily dispersed seeds, well‐protected seeds, and long‐term seed dormancy are best suited to establish in bracken.

### 4.2 Processes

Our finding that seed rain in bracken is only around 59% of that in the nearby forest reflects the generally low seed rain found by other studies in tropical rain forests in open areas adjacent to the forest. For instance, in Ngel Nyaki Forest, Nigeria, Barnes and Chapman (2014) found that seed rain in the grassland area 20–30 m from the forest edge was only 11% of that from the nearby forest while in Kibale forest in Uganda Duncan and Chapman (1999) found that the area dominated by *Pennisetum purpureum* Schumach. received roughly 1% of the seed rain about 100 m from the forest. This indicates dispersal limitation—a situation likely exacerbated by few effective dispersal agents such as frugivorous birds and bats using these areas and the low availability of perches for them (*sensu* Saavedra et al., 2015). We note that *Clutia abyssinica*, the only species whose seedlings showed a clear tendency to favor bracken, was not detected in the seed rain. This could have resulted from our seed traps being too high above the ground or too far from any plants to receive the ballistic‐dispersed seeds from this short (occasionally to 5 m) multi‐stemmed woody plant. Although its seeds are relatively small (seed size = 2.7 mm), we expected to find them in the seed rain as smaller seeds like those of *Allophylus abyssinicus* (seed size = 2.6 mm) and *Nuxia congesta* (seed size = 1.5 mm) were detected.

The density of viable seeds in the soil was generally low and had little discernible relationship with regeneration differences between forest and bracken. Our overall seed bank density of about 300 seeds/m^2^ is about half the 500–600 seeds/m^2^ found in later successional stages and less than a third of the 1,000–2,700 seeds/m^2^ found previously in the early stages of succession following cultivation in three montane forests of Bwindi, Mgahinga, and Echuya (Karlowski, 2006). This may be due to the difference in methods (we likely lost many seeds smaller than 2 mm during washing and Karlowski (2006) did not target areas in or next to bracken). The limited seed bank may indicate that few species possess long‐term seed dormancy. Previous studies in East and Southern Africa have found that many trees including *Croton macrostachuys*,* Prunus africana,* and *Symphonia globulifera* have recalcitrant seeds (Mng'omba, du Toit, & Akinnifesi, 2007; Muhanguzi, Obua, & Oryem‐Origa, 2002; Negash, 2003, 2004; Shehaghilo, 1989; Wakjira & Negash, 2013). In addition, the low abundance of tree seeds in deforested areas has been reported to limit forest recovery elsewhere in the African tropics (Hall & Swaine, 1980; Mukhongo, Kinyamario, Chira, & Musila, 2011; Teketay & Granstrom, 1995). As such, the relative numbers provided by our data suggest that the soil seed bank has little apparent role or contribution to forest recovery in bracken‐dominated clearings, although *Clutia abyssinica* as the only species whose seedlings favor bracken is an exception. The large number and diversity of seeds from nonwoody plants in the seed bank under bracken was unexpected (5,905 of 6,953 = 85% and 20 species). The implications of this are unclear, but it may be that when opportunities for germination arise, the ensuing competition is intense and contributes further to impeding woody regrowth. Further study would be required to evaluate this mechanism.

We also found that tree seed removal in bracken over a 3‐day interval was about 173% of that in the nearby forest. Compounding this removal over a longer period shows that this differential process is adequate to explain marked differences between the forest and bracken. For example, even if forest and bracken started with the same seed number, maintaining the same 3‐day rate of seed removal over a 30‐day interval would result in more than 800 times more seeds in the forest. Other studies have also reported a significantly higher rate of seed removal in treeless areas than in nearby forest (in Indonesia, Blackham & Corlett, 2015; in Netherlands, den Ouden, 2000; and in Madagascar, Razafindratsima, 2017) although such patterns are not universal, for example, grassland versus forest in Kibale Forest in Uganda (Ssekuubwa, Loe, Sheil, Tweheyo, & Moe, in press).

The rapid removal of the grain crops compared to the native trees shows that selective factors are involved. A recent study in Lake Mburo National Park in Uganda also found that conspicuous and palatable seeds, including crop species, are more liable to removal than native trees (Acanakwo, Sheil, & Moe, 2017). In our study, we found that the conspicuous and less well‐protected tree species *Strombosia scheffleri* (seed size = 23.7 mm) and *Chrysophyllum pruniforme* (seed size = 19.0 mm) had higher seed removal rates per 3 days in bracken and the forest than *Faurea saligna* (seed size = 1.1 mm), *Neoboutonia macrocalyx* (seed size = 7.6 mm), and *Olinia rochetiana* (seed size = 10.0 mm) which are protected by hard seed coats. When ranked *Strombosia scheffleri* had the highest removal rates in both bracken and forest and was consistently followed by *Chrysophyllum pruniforme*,* Faurea saligna*,* Olinia rochetiana,* and *Neoboutonia macrocalyx* (see Figure 3).

Our rodent capture rate was five times higher in bracken than in the forest. Dense cover in bracken provides good cover for rodents. A comparable study in Netherlands estimated that rodent density was four times higher in bracken than in the neighboring forest (den Ouden, 2000), and many previous studies in tropical African forests have reported significantly higher rodent densities in forest clearings than in the continuous forest (in Uganda, Isabirye‐Basuta & Kasenene, 1987; in Ghana, Jeffrey, 1977; in Madagascar, Razafindratsima, 2017) although some studies have failed to find significant differences (e.g., in Tanzania, Cordeiro et al., 2009). Capture rates in our study, that is, 26 rodents per 100 trapping sessions, are similar to those found by one previous study in Bwindi and relatively high compared to various other tropical African forests. For example, in Bwindi, Kasangaki et al. (2003) found 36 rodents per 100 trap nights although Mawanda (2014) who worked on the forest edge caught only five rodents per 100 trap nights. In the East Usambara Mountains of Tanzania, Cordeiro et al. (2009) found about four rodents per 100 trap nights in the fragmented area and about five rodents per 100 trap nights in the continuous forest. We note, however, that there can be considerable local variation, for example, in Kibale Forest Isabirye‐Basuta and Kasenene (1987) who do not quote trapping numbers directly, report 0.7–26 rodents/ha in a deforested area and 0.7–21.3/ha in mature forest. In addition, densities may vary considerably over time as seen in other regions, although there has been little attention to such variation in the wet tropics (Adler, 1994; Korpimäki, Brown, Jacob, & Pech, 2004; Krebs, 1996).

The most common rodents in bracken and at the forest edge (i.e., *Lophuromys* spp and *Hybomys univittatus*) are large and omnivorous implying that they opportunistically consume a range of food items including leaves, seeds, roots, insects, and small vertebrates (Kingdon, 1997). Despite some limitations with the camera traps, we still detected several larger rodents, too big for our traps, known for scatter‐hoarding including the Forest giant pouched rat *Cricetomys emini* Wroughton and Carruther's mountain squirrel *Funisciurus carruthersi* Thomas (Appendix S5). While we expect that most seeds are consumed, the presence of species known for seed caching also raises the question of seed fate and whether these rodents move these seeds toward or away from the adjacent forest. In suitable contexts, rodents can aid (rather than impede) seed arrival (as found for *Carapa* spp. seeds in Nyungwe by Nyiramana, Mendoza, Kaplin, & Forget, 2011). Nonetheless, our data suggest that bracken may impact neighboring vegetation, at least up to 25 meters, through the influence on rodents and related seed removal.

This is the first study in the African tropics that simultaneously examines establishment limitation and key recruitment determinants (seed rain, soil seed bank, seed survival, and rodent densities) in both forest and bracken‐dominated areas. We provide evidence of low seed rain and high rates of seed removal in bracken. Our previous observations have suggested that various additional factors, including shade, litter, and fire, may also contribute to the poor regeneration of woody plants in bracken clearings in Bwindi (Ssali et al., 2017). However, if seed arrival and removal are the primary limits to tree regeneration, we would predict that adding seeds and excluding rodents would bolster forest recovery in these bracken‐dominated clearings.

## CONFLICT OF INTEREST

None declared.

## AUTHORS’ CONTRIBUTIONS

FS, SRM, and DS designed the study. FS carried out fieldwork and data analysis in collaboration with SRM and DS. FS led the writing with input from DS and SRM.
