# Supplementary material for: Tree seed rain and seed removal, but not the seed bank, impede forest recovery in bracken (Pteridium aquilinum (L.) Kuhn)‐dominated clearings in the African highlands
Source: Ecol Evol. 2018 Apr 2;8(8):4224–36. doi: 10.1002/ece3.3944 (PMC5916285; doi:10.1002/ece3.3944)
Supplement: Supplementary file 1 [file ECE3-8-4224-s001.docx]

**Appendix 1**: List of species in the forest (FO), the edge (ED) and bracken (BR) showing the abundance of their seed rain, soil seed bank and seedling population. While woody species were recorded in all the studies, non-woody species were only identified in the soil seed bank study.

|  | Seed rain | | |  | Soil seed bank | | |  | Small seedlings | | |  | Large seedlings | | |
| --- | --- | --- | --- | --- | --- | --- | --- | --- | --- | --- | --- | --- | --- | --- | --- |
|  | FO | ED | BR |  | FO | ED | BR |  | FO | ED | BR |  | FO | ED | BR |
| N sampling points | 40 | 30 | 40 |  | 10 | 10 | 10 |  | 40 | 40 | 40 |  | 40 | 40 | 40 |
| Species |  |  |  |  |  |  |  |  |  |  |  |  |  |  |  |
| Trees |  |  |  |  |  |  |  |  |  |  |  |  |  |  |  |
| *Faurea saligna* Harv. | 2392 | 454 | 447 |  | 0 | 0 | 0 |  | 244 | 145 | 265 |  | 0 | 1 | 0 |
| *Nuxia congesta* R.Br. ex Fresen. | 939 | 1481 | 2346 |  | 3 | 2 | 1 |  | 0 | 1 | 15 |  | 0 | 3 | 5 |
| *Ficalhoa laurifolia* Hiern | 859 | 1659 | 55 |  | 0 | 0 | 0 |  | 0 | 0 | 0 |  | 3 | 0 | 0 |
| *Allophylus abyssinicus* (Hochst.) Radlk. | 303 | 69 | 2 |  | 4 | 0 | 0 |  | 129 | 14 | 3 |  | 6 | 3 | 0 |
| *Dombeya torrida* (J.F.Gmel.) Bamps | 170 | 0 | 0 |  | 1 | 0 | 0 |  | 0 | 0 | 0 |  | 0 | 0 | 0 |
| *Polyscias fulva* (Hiern) Harms | 159 | 16 | 0 |  | 0 | 0 | 0 |  | 0 | 3 | 0 |  | 0 | 0 | 0 |
| *Morella salicifolia* (Hochst. ex A.Rich.) Verdc. & Polhill | 100 | 140 | 1 |  | 0 | 0 | 0 |  | 0 | 0 | 0 |  | 0 | 0 | 0 |
| *Neoboutonia macrocalyx* Pax | 72 | 2 | 0 |  | 0 | 0 | 0 |  | 0 | 0 | 0 |  | 5 | 1 | 0 |
| *Ficus densistipulata* De Wild. | 64 | 214 | 0 |  | 0 | 0 | 0 |  | 0 | 0 | 0 |  | 0 | 1 | 0 |
| *Croton macrostachyus* Hochst. ex Delile | 62 | 1 | 1 |  | 1 | 0 | 0 |  | 0 | 16 | 10 |  | 0 | 0 | 0 |
| *Rytigynia ruwenzoriensis* (De Wild.) Robyns | 52 | 5 | 33 |  | 1 | 0 | 0 |  | 370 | 650 | 26 |  | 59 | 9 | 3 |
| *Psychotria mahonii* C.H.Wright | 47 | 66 | 245 |  | 2 | 1 | 0 |  | 281 | 135 | 115 |  | 6 | 3 | 1 |
| *Chrysophyllum pruniforme* Pierre ex Engl. | 32 | 8 | 2 |  | 0 | 0 | 0 |  | 41 | 46 | 0 |  | 12 | 3 | 0 |
| *Ilex mitis* (L.) Radlk. | 28 | 0 | 0 |  | 2 | 1 | 0 |  | 0 | 1 | 0 |  | 0 | 0 | 0 |
| *Xymalos monospora* (Harv.) Baill. | 21 | 2 | 0 |  | 0 | 0 | 0 |  | 68 | 8 | 0 |  | 5 | 4 | 0 |
| *Agauria salicifolia* (Comm. ex Lam.) Hook.f. ex Oliv. | 14 | 0 | 0 |  | 0 | 0 | 0 |  | 0 | 0 | 0 |  | 0 | 0 | 0 |
| *Olinia rochetiana* Juss. | 13 | 4 | 0 |  | 1 | 0 | 0 |  | 0 | 0 | 0 |  | 0 | 0 | 0 |
| *Macaranga capensis* (Baill.) Sim | 11 | 6 | 5 |  | 5 | 4 | 5 |  | 14 | 12 | 3 |  | 8 | 6 | 1 |
| *Podocarpus latifolius* (Thunb.) R.Br. ex Mirb. | 9 | 5 | 0 |  | 0 | 0 | 0 |  | 6 | 2 | 0 |  | 17 | 0 | 0 |
| *Rytigynia kigeziensis* Verdc. | 7 | 0 | 1 |  | 0 | 0 | 0 |  | 0 | 0 | 0 |  | 1 | 0 | 0 |
| *Olea capensis* L. | 6 | 3 | 2 |  | 0 | 0 | 0 |  | 3 | 4 | 0 |  | 11 | 0 | 0 |
| *Ekebergia capensis* Sparrm. | 6 | 4 | 0 |  | 0 | 0 | 0 |  | 17 | 13 | 1 |  | 1 | 0 | 0 |
| *Vepris nobilis* (Delile) Mziray | 4 | 1 | 0 |  | 0 | 0 | 0 |  | 30 | 4 | 1 |  | 25 | 2 | 0 |
| *Maesa lanceolata* Forssk. | 3 | 17 | 57 |  | 88 | 59 | 113 |  | 0 | 2 | 3 |  | 0 | 1 | 0 |
| *Rapanea melanophloeos* (L.) Mez | 3 | 3 | 2 |  | 0 | 3 | 1 |  | 4 | 5 | 1 |  | 1 | 0 | 0 |
| *Bridelia micrantha* (Hochst.) Baill. | 3 | 6 | 1 |  | 0 | 0 | 0 |  | 0 | 0 | 0 |  | 0 | 0 | 0 |
| *Pittosporum viridiflorum* Sims | 1 | 0 | 1 |  | 0 | 0 | 0 |  | 1 | 0 | 0 |  | 0 | 0 | 0 |
| *Alangium chinense* (Lour.) Harms | 1 | 0 | 0 |  | 0 | 0 | 0 |  | 8 | 4 | 2 |  | 1 | 0 | 0 |
| *Strombosia scheffleri* Engl. | 0 | 0 | 2 |  | 0 | 0 | 0 |  | 14 | 0 | 0 |  | 0 | 0 | 1 |
| *Dodonaea viscosa* Jacq. | 0 | 0 | 1 |  | 0 | 0 | 0 |  | 0 | 0 | 2 |  | 0 | 0 | 0 |
| *Syzygium guineense* (Willd.) DC. | 0 | 3 | 0 |  | 0 | 0 | 0 |  | 15 | 2 | 0 |  | 2 | 0 | 0 |
| *Gymnosporia acuminata* (L.f.) Szyszyl. | 0 | 0 | 0 |  | 0 | 4 | 0 |  | 5 | 2 | 1 |  | 15 | 5 | 0 |
| *Zanthoxylum gilletii* (De Wild.) P.G.Waterman | 0 | 0 | 0 |  | 1 | 0 | 1 |  | 1 | 1 | 1 |  | 0 | 0 | 0 |
| *Peddiea fischeri* Engl. | 0 | 0 | 0 |  | 3 | 1 | 1 |  | 0 | 0 | 0 |  | 0 | 0 | 0 |
| *Allophylus africanus* (Gilg) Verdc. | 0 | 0 | 0 |  | 0 | 0 | 0 |  | 2 | 0 | 0 |  | 7 | 0 | 0 |
| *Albizia gummifera* (J.F.Gmel.) C.A.Sm. | 0 | 0 | 0 |  | 0 | 0 | 0 |  | 1 | 0 | 0 |  | 1 | 0 | 0 |
| *Alchornea hirtella* Benth. | 0 | 0 | 0 |  | 0 | 0 | 0 |  | 1 | 0 | 0 |  | 0 | 0 | 0 |
| *Bersama abyssinica* Fresen. | 0 | 0 | 0 |  | 0 | 0 | 0 |  | 4 | 2 | 0 |  | 0 | 0 | 0 |
| *Cassipourea gummiflua* Tul. | 0 | 0 | 0 |  | 0 | 0 | 0 |  | 0 | 1 | 9 |  | 1 | 2 | 0 |
| *Connarus longistipitatus* Gilg | 0 | 0 | 0 |  | 0 | 0 | 0 |  | 4 | 3 | 0 |  | 2 | 3 | 0 |
| *Drypetes gerrardii* Hutch. | 0 | 0 | 0 |  | 0 | 0 | 0 |  | 1 | 2 | 0 |  | 6 | 2 | 0 |
| *Erythrococca trichogyne* (Müll.Arg.) Prain | 0 | 0 | 0 |  | 0 | 0 | 0 |  | 8 | 3 | 0 |  | 11 | 6 | 0 |
| *Ficus asperifolia* Miq. | 0 | 0 | 0 |  | 0 | 0 | 0 |  | 0 | 0 | 1 |  | 0 | 0 | 0 |
| *Galiniera saxifraga* (Hochst.) Bridson | 0 | 0 | 0 |  | 0 | 0 | 0 |  | 0 | 0 | 0 |  | 2 | 1 | 0 |
| *Heinsenia diervilleoides* K.Schum. | 0 | 0 | 0 |  | 0 | 0 | 0 |  | 0 | 1 | 0 |  | 0 | 3 | 0 |
| *Mystroxylon aethiopicum* (Thunb.) Loes. | 0 | 0 | 0 |  | 0 | 0 | 0 |  | 2 | 1 | 0 |  | 2 | 0 | 0 |
| *Ficus exasperata* Vahl | 0 | 0 | 0 |  | 0 | 0 | 0 |  | 0 | 0 | 0 |  | 1 | 0 | 0 |
| *Oxyanthus speciosus* DC. | 0 | 0 | 0 |  | 0 | 0 | 0 |  | 4 | 0 | 0 |  | 8 | 1 | 0 |
| *Psychotria kirkii* Hiern | 0 | 0 | 0 |  | 0 | 0 | 0 |  | 12 | 0 | 0 |  | 4 | 2 | 0 |
| *Pauridiantha callicarpoides* (Hiern) Bremek. | 0 | 0 | 0 |  | 0 | 0 | 0 |  | 0 | 0 | 0 |  | 1 | 0 | 0 |
| *Prunus africana* (Hook.f.) Kalkman | 0 | 0 | 0 |  | 0 | 0 | 0 |  | 8 | 4 | 0 |  | 0 | 0 | 0 |
| *Rytigynia beniensis* (De Wild.) Robyns | 0 | 0 | 0 |  | 0 | 0 | 0 |  | 1 | 0 | 0 |  | 0 | 2 | 1 |
| *Rytigynia bugoyensis* (K.Krause) Verdc. | 0 | 0 | 0 |  | 0 | 0 | 0 |  | 0 | 0 | 0 |  | 5 | 1 | 0 |
| *Ritchiea albersii* Gilg | 0 | 0 | 0 |  | 0 | 0 | 0 |  | 4 | 5 | 0 |  | 1 | 2 | 0 |
| Unidentified 1 | 0 | 0 | 0 |  | 0 | 0 | 0 |  | 2 | 0 | 0 |  | 0 | 0 | 0 |
| Unidentified 2 | 0 | 0 | 0 |  | 0 | 0 | 0 |  | 0 | 0 | 0 |  | 1 | 0 | 0 |
| Treelets and woody climbers |  |  |  |  |  |  |  |  |  |  |  |  |  |  |  |
| *Clutia abyssinica* Jaub. & Spach | 0 | 0 | 0 |  | 0 | 5 | 3 |  | 16 | 125 | 13 |  | 16 | 39 | 29 |
| *Clerodendrum johnstonii* Oliv. | 12 | 15 | 0 |  | 0 | 0 | 0 |  | – | – | – |  | – | – | – |
| *Englerina woodfordioides* (Schweinf.) Balle*** | 3 | 0 | 0 |  | 0 | 0 | 0 |  | – | – | – |  | – | – | – |
| *Clematis* spp | 0 | 1 | 0 |  | 0 | 1 | 0 |  | – | – | – |  | – | – | – |
| *Adenia* spp | 0 | 0 | 0 |  | 2 | 3 | 0 |  | – | – | – |  | – | – | – |
| *Dregea schimperi* (Decne.) Bullock | 0 | 0 | 0 |  | 0 | 1 | 0 |  | – | – | – |  | – | – | – |
| *Mikania cordata* (Burm.f.) B.L.Rob. | 0 | 0 | 0 |  | 31 | 51 | 19 |  | – | – | – |  | – | – | – |
| *Solanecio mannii* (Hook.f.) C.Jeffrey | 0 | 0 | 0 |  | 1 | 0 | 0 |  | – | – | – |  | – | – | – |
| *Salacia elegans* Welw. ex Oliv. | 55 | 15 | 1 |  | 0 | 0 | 0 |  | – | – | – |  | – | – | – |
| *Keetia gueinzii* (Sond.) Bridson | 48 | 0 | 0 |  | 0 | 0 | 0 |  | – | – | – |  | – | – | – |
| *Landolphia* spp | 16 | 2 | 0 |  | 0 | 0 | 0 |  | – | – | – |  | – | – | – |
| *Rutidea orientalis* Bridson | 14 | 8 | 1 |  | 0 | 0 | 0 |  | – | – | – |  | – | – | – |
| *Vernonia auriculifera* Hiern. | 0 | 0 | 0 |  | 0 | 0 | 4 |  | – | – | – |  | – | – | – |
| *Vernonia kirungae* R.E.Fr. | 0 | 36 | 0 |  | 0 | 5 | 0 |  | – | – | – |  | – | – | – |
| *Senecio* spp | 0 | 0 | 0 |  | 24 | 44 | 27 |  | – | – | – |  | – | – | – |
| Non-woody plants |  |  |  |  |  |  |  |  |  |  |  |  |  |  |  |
| *Basella alba* L. | – | – | – |  | 1 | 0 | 1 |  | – | – | – |  | – | – | – |
| *Ipomoea involucrata* P.Beauv. | – | – | – |  | 1 | 0 | 0 |  | – | – | – |  | – | – | – |
| *Plectranthus* sp | – | – | – |  | 2 | 5 | 106 |  | – | – | – |  | – | – | – |
| *Asplenium* sp | – | – | – |  | 0 | 0 | 1 |  | – | – | – |  | – | – | – |
| *Achyranthes aspera* L. | – | – | – |  | 2 | 1 | 11 |  | – | – | – |  | – | – | – |
| *Brachystephanus africanus* S. Moore | – | – | – |  | 2 | 17 | 0 |  | – | – | – |  | – | – | – |
| *Commelina* sp | – | – | – |  | 1 | 2 | 7 |  | – | – | – |  | – | – | – |
| *Conyza bonariensis* (L.) Cronquist | – | – | – |  | 2 | 4 | 3 |  | – | – | – |  | – | – | – |
| *Crassocephalum montuosum* (S.Moore) Milne-Redh. | – | – | – |  | 18 | 38 | 19 |  | – | – | – |  | – | – | – |
| *Cyathula uncimulata* (Schrad.) Schinz | – | – | – |  | 1 | 3 | 0 |  | – | – | – |  | – | – | – |
| *Droguetia iners* (Forssk.) Schweinf. | – | – | – |  | 0 | 1 | 0 |  | – | – | – |  | – | – | – |
| *Drymaria cordata* (L.) Willd. ex Roem. & Schult. | – | – | – |  | 6 | 5 | 20 |  | – | – | – |  | – | – | – |
| *Helichrysum* sp | – | – | – |  | 2 | 1 | 0 |  | – | – | – |  | – | – | – |
| *Impatiens* sp | – | – | – |  | 2 | 0 | 4 |  | – | – | – |  | – | – | – |
| *Kalanchoe densiflora* Rolfe | – | – | – |  | 1 | 0 | 0 |  | – | – | – |  | – | – | – |
| *Physalis peruviana* L. | – | – | – |  | 3 | 2 | 6 |  | – | – | – |  | – | – | – |
| *Pilea holstii* Engl. | – | – | – |  | 9 | 3 | 23 |  | – | – | – |  | – | – | – |
| *Pycnostachys elliotii* S.Moore | – | – | – |  | 22 | 17 | 20 |  | – | – | – |  | – | – | – |
| *Solanum nigrum* L. | – | – | – |  | 0 | 4 | 8 |  | – | – | – |  | – | – | – |
| *Spermacoce princeae* (K.Schum.) Verdc. | – | – | – |  | 62 | 11 | 52 |  | – | – | – |  | – | – | – |
| *Cyperus* sp | – | – | – |  | 23 | 159 | 166 |  | – | – | – |  | – | – | – |
| *Oplismenus hirtellus* (L.) P.Beauv. | – | – | – |  | 0 | 23 | 842 |  | – | – | – |  | – | – | – |
| *Panicum* sp | – | – | – |  | 46 | 92 | 99 |  | – | – | – |  | – | – | – |
| *Setaria* sp | – | – | – |  | 17 | 31 | 55 |  | – | – | – |  | – | – | – |
| *Mimulopsis solmsii* Schweinf. | – | – | – |  | 2 | 5 | 0 |  | – | – | – |  | – | – | – |
| *Rubus apetalus* Poir. | – | – | – |  | 7 | 14 | 3 |  | – | – | – |  | – | – | – |
| *Triumfetta cordifolia* A.Rich. | – | – | – |  | 1 | 6 | 4 |  | – | – | – |  | – | – | – |
| *Lobelia stuhlmannii* Schweinf. ex Stuhlm. | – | – | – |  | 6 | 7 | 6 |  | – | – | – |  | – | – | – |
| *Pavonia urens* Cav. | – | – | – |  | 1 | 0 | 0 |  | – | – | – |  | – | – | – |
| **Unidentified** | 7 | 5 | 4 |  | 293 | 93 | 112 |  | – | – | – |  | – | – | – |
| **Total for identified woody species** | 5536 | 4251 | 3210 |  | 170 | 185 | 175 |  | 1320 | 1218 | 462 |  | 247 | 105 | 41 |

Note: * is a mistletoe

**Appendix 2:** Kendall’s correlations (n= 11) between seed density and distance from the forest interior into bracken-dominated clearings for the 16 most common woody species. Significant values (P < 0.05) are denoted in bold.

| Species | τ | P |
| --- | --- | --- |
| *Ficalhoa laurifolia* Hiern | -0.23 | 0.359 |
| *Faurea saligna* Harv. | -0.75 | **0.001** |
| *Nuxia congesta* R.Br. ex Fresen. | 0.42 | 0.087 |
| *Polyscias fulva* (Hiern) Harms | -0.58 | **0.02** |
| *Allophylus abyssinicus* (Hochst.) Radlk. | -0.79 | **0.001** |
| *Psychotria mahonii* C.H.Wright | 0.02 | 0.938 |
| *Chrysophyllum pruniforme* Pierre ex Engl. | -0.77 | **0.002** |
| *Rutidea orientalis* Bridson | -0.49 | 0.068 |
| *Croton macrostachyus* Hochst. ex Delile | -0.65 | **0.012** |
| *Salacia elegans* Welw. ex Oliv. | -0.64 | **0.01** |
| *Dombeya torrida* (J.F.Gmel.) Bamps | -0.79 | **0.002** |
| *Ficus densistipulata* De Wild. | -0.18 | 0.466 |
| *Rytigynia ruwenzoriensis* (De Wild.) Robyns | -0.37 | 0.126 |
| *Keetia gueinzii* (Sond.) Bridson | -0.65 | **0.012** |
| *Maesa lanceolata* Forssk. | -0.18 | 0.483 |
| *Neoboutonia macrocalyx* Pax | -0.73 | **0.005** |

**Appendix 3:** Seed rain density (seeds m^-2^ yr^-1^ ± 1 SE) of each of 16 common woody species plotted relative to the edge between the forest and bracken with the forest dark grey, the edge grey and the bracken-dominated area unshaded.


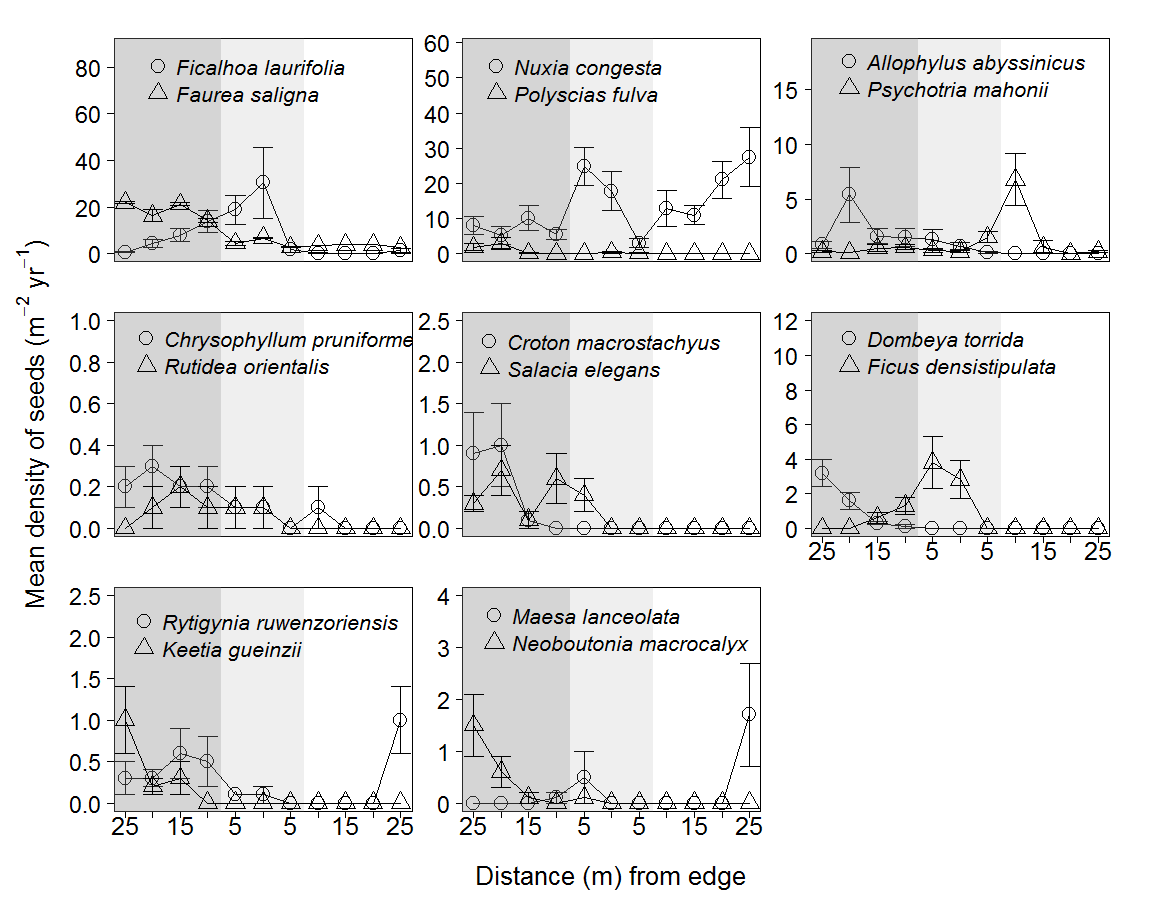


**Appendix 4.** Number of rodent images and proportion of seeds removed in seven selected sites in the forest, edge and bracken within three days. The superscripts a, b and c denote bracken, edge and forest, respectively.

|  |  | *N rodent images* | | |  | *N all images* | | |  | Seeds removed (%) | | |
| --- | --- | --- | --- | --- | --- | --- | --- | --- | --- | --- | --- | --- |
|  |  | Forest | Edge | Bracken |  | Forest | Edge | Bracken |  | Forest | Edge | Bracken |
| *N cameras over 3 days* |  | 5 | 4 | 5 |  | 5 | 4 | 5 |  |  |  |  |
| *Transects (UTM Zone 35 coordinates)* | *Bracken patch size (ha)* |  |  |  |  |  |  |  |  |  |  |  |
| 0807885, 9884441^a^  0807908, 9884435^b^  0807922, 9884441^c^ | 0.41 | 0 | – | 2 |  | 3 | – | 44 |  | 7.7 | – | 69.2 |
| 0808175, 9883895^a^  0808175, 9883878^b^  0808173, 9883847^c^ | 0.32 | 19 | 5 | – |  | 76 | 23 | – |  | 38.5 | 38.5 | – |
| 0808485, 9884142^a^  0808460, 9884143^b^  0808451, 9884123^c^ | 0.35 | 16 | – | 2 |  | 69 | – | 23 |  | 61.5 | – | 100 |
| 0808902, 9882860^a^  0808876, 9882862^b^  0808857, 9882853^c^ | 1.4 | – | 3 | 26 |  | – | 36 | 62 |  | – | 84.6 | 92.3 |
| 0808499, 9882840^a^  0808480, 9882833^b^  0808460, 9882813^c^ | 2.5 | 12 | – | 2 |  | 19 | – | 58 |  | 46.2 | – | 53.8 |
| 0808413, 9882735^a^  0808410, 9882761^b^  0808414, 9882786^c^ | 0.18 | – | 15 | 8 |  | – | 43 | 15 |  | – | 76.9 | 69.2 |
| 0808291, 9884499^a^  0808262, 9884509^b^  0808241, 9884505^c^ | 0.08 | 15 | 17 | – |  | 44 | 29 | – |  | 61.5 | 84.6 | – |
| 0809768, 9883123^a^  0809787, 9883137^b^  0809813, 9883152^c^ | 0.3 | – | – | – |  | – | – | – |  | – | – | – |
| 0809713, 9883098^a^  0809700, 9883073^b^  0809702, 9883054^c^ | 0.18 | – | – | – |  | – | – | – |  | – | – | – |
| 0809646, 9883047^a^  0809642, 9883069^b^  0809640, 9883101^c^ | 0.15 | – | – | – |  | – | – | – |  | – | – | – |
| Average (± 1 SE) |  | 12.4 ± 3.3 | 10 ± 3.5 | 8 ± 4.7 |  | 42.2 ± 14.0 | 32.8 ± 4.3 | 40.4 ± 9.3 |  | 43.1 ± 9.9 | 71.2 ± 11.0 | 76.9 ± 8.4 |

**Appendix 5**. Automatic camera trap images of common rodents in bracken (a), at the edge (b) and in the surrounding forest (c - d). The rodent species are a) *Hybomys univittatus*, b) *Praomys* spp, c) *Funisciurus carruthersi* and d) *Cricetomys emini*.


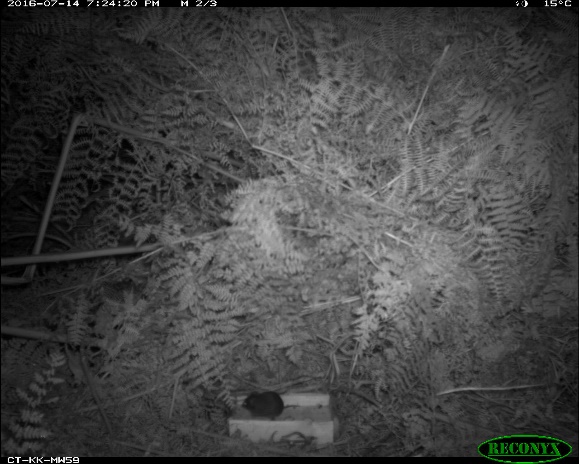
**
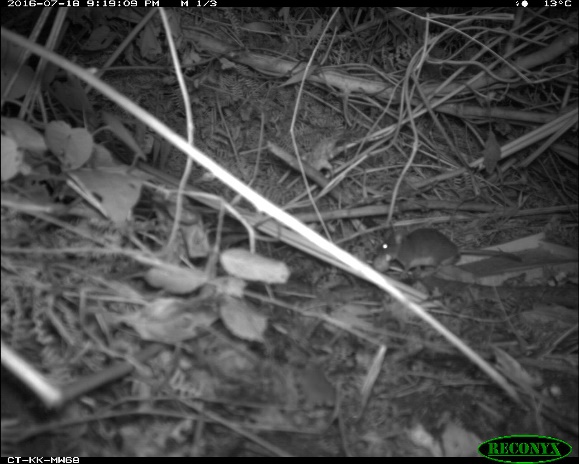

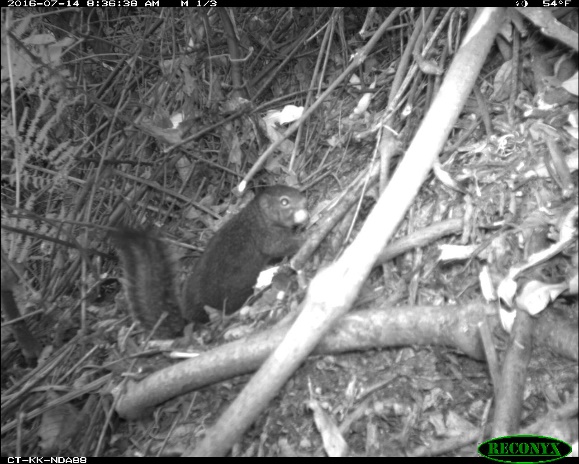
**
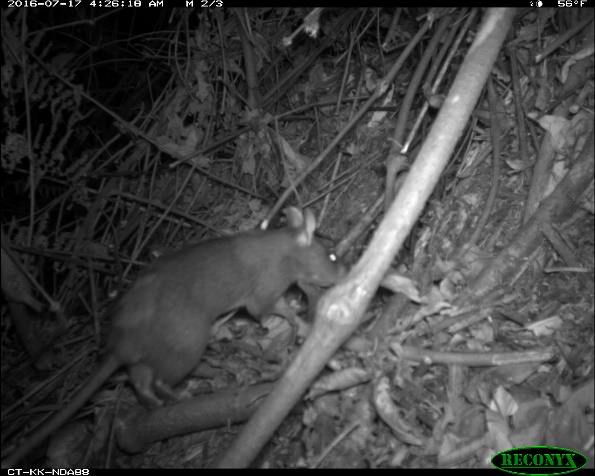


a)

b)

c)

d)
